# A case report of an unusual firearm injury: projectile lodged between thoracic aorta and vertebral column

**DOI:** 10.1186/1749-8090-8-S1-P28

**Published:** 2013-09-11

**Authors:** J Konecny, A Klvacek, P Santavy, V Lonsky, M Simek

**Affiliations:** 1 Cardiac Surgery Department, University Hospital of Olomouc, Olomouc, Czech Republic

## Background

Firearm – related injuries of the thorax and heart are a major problem worldwide. Mortality rate in non-cardiac thoracic injuries is very low compared with that of cardiac injury. Because of the complexity of the injury a gunshot wound to the heart has the highest mortality rate. The rate of thoracic aorta injuries is burdened with a high mortality (80-90%) and management of this injury is very difficult.

## Methods

We present a case of a 45 year old inebriated man, who had a gunshot injury to the chest by an air gun. He was taken to the emergency department immediately after the incident. Physical examination revealed one entrance gunshot wound 2 cm above the left nipple. A thoracic CT scan showed the position of the projectile between the thoracic aorta and body of vertebra Th 11 but the trajectory of the projectile was obscure. There was no pneumothorax, fluidothorax, fluidopericardium, or extravasation. The patient was admitted for observation to The Cardiovascular department. During the whole entire hospitalization he was hemodynamically stable and the position of the projectile was stable. He was administered the antibiotic Zinnat for 10 days as prophylaxis. After 5 days of hospitalization the patient was discharged to home-care.

## Results

Routine check-ups at the surgical department and every half year a thoracic CT scan were recommended because of the spontaneously changing projectile position.

## Conclusions

Thoracic penetrating wounds are frequently encountered in urban medical centers. The case of a similar projectile trajectory wasn´t described in any literature. Because of the complicated projectile position surgical removal was burdened by a high mortality risk. There is also the possibility that spontaneous movement of the projectile could harm the thoracic aorta. That is periodical observation of the patient was recommended.

